# Supplementary material for: A recipe for systems change: Predictive modeling and street-level bureaucracy among homeless services
Source: PLoS One. 2025 Aug 21;20(8):e0328822. doi: 10.1371/journal.pone.0328822 (PMC12370027; doi:10.1371/journal.pone.0328822)
Supplement: S1 File — Tessellations [42] and other geometric insights are used to stress hierarchical subtleties. (DOCX) [file pone.0328822.s001.docx]

***Appendix A: Commentary from workers***

As we have established, there is a litany of work devoted to measuring Lipsky’s theories on street-level bureaucracy using qualitative methods, with which we have drawn upon to inform our study [27,28,55,4,65]. In a similar vein, we now turn to reflective commentary from workers in this study to reinforce the legitimacy of our quantitative findings.

Three months after each 100-Day Challenge*™*, RE!NSTITUTE™ follows up with the participating agency. They are asked for general feedback. When asked if “There is evidence of successful collaboration between agencies/orgs and/or sectors 3 months after the 100-Day Challenge*™*: 38% Strongly Agree, 47% Agree, 14.3% Neutral, and 0% said “no.” Here are a collection of additional sentiments that agencies shared by writing in comments to RE!NSTITUTE™:

“Collaboration was key. It is rare to have space where there are direct service providers and people with a lot of positional authority having conversations together across communities. That is very positive. They really have achieved the aim of not having people go back to homelessness from temporary covid response housing. At least two thirds going into housing. Very significant.”

“Collaboration was huge - have never been part of something in which so many people from different parts of the community came together.”

“What was sustained were relationships that kept going, ongoing conversations etc.”

“What was impressive was to see and meet dedicated navigators and problem solvers that were really connected to the participants. The 100-Day Challenge*™* really opened my eyes. It allowed me to see more. Also saw that we don't have enough resources - not enough housing units.”

“Expanded service delivery within our two departments. Direct engagement, navigation, outreach - some of those things ended up adapting to and learning because of the collaboration”

***Appendix B: Quasi periodic patterns as potential visualization replacements***

We observe that each sub-triangle representing one brand of innovation (either on the conceptual front, revealed through Figure 1, or on the realized side, shown through Figure 3) has the opportunity of appearing only once. This is due to the nature of the construction. With the base of a triangle revealing how common changes in that innovation category is and the altitude showing our confidence in those estimates, comparing the shapes and sizes of similar looking triangles becomes potentially problematic if an analyst observes each shape once, as in Figure 3. The problem gets aggravated especially if (like “policy” and “resources” on Figure 3) the triangles are not adjacent. In this section, we offer a system to remedy this by using the principle of repetition. Figure 4 below describes the intricacies.

Each triangle is subdivided many times, each time respecting its original shape, so no new information not present in our gathered data can creep in, and the resulting fragments are organized in a circular fashion, spiraling inwards. The main benefit is an amplifying minor differences in the original triangles: a viewer observing similar shapes being repeated many times will be able to register and retain smaller differences between similar shapes (like “policy” and “resources”) again, through experiencing these differences *many* times - more solidly than they would have if they saw these shapes once (please see Figure 3). These circular shapes bring out quasi-periodic patterns, that is patterns which are self-similar but non periodic. The ordered (that is self-similar) aspect helps one experience the benefit of repetition, while the chaotic (that is the nonperiodic) aspect helps one retain interest, despite that repetition. The patterns originate from group theory and non-commutative geometry and are special cases of Penrose tiles^^[[1]](#footnote-1)^^.


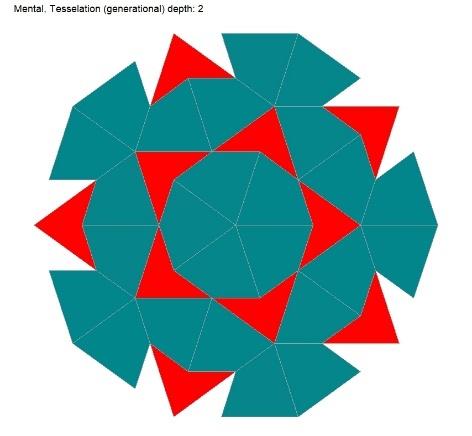
**
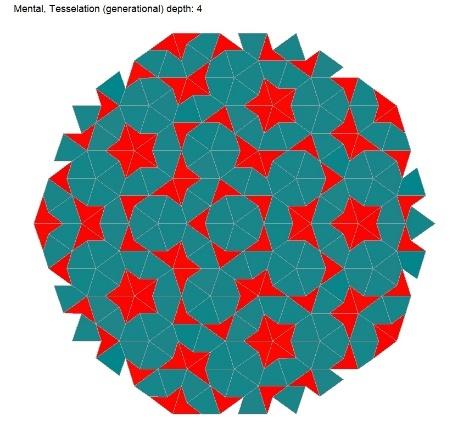
**
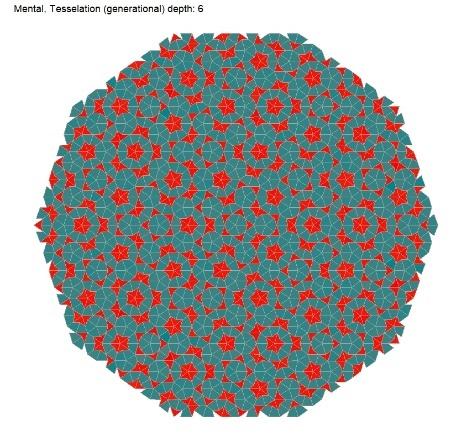


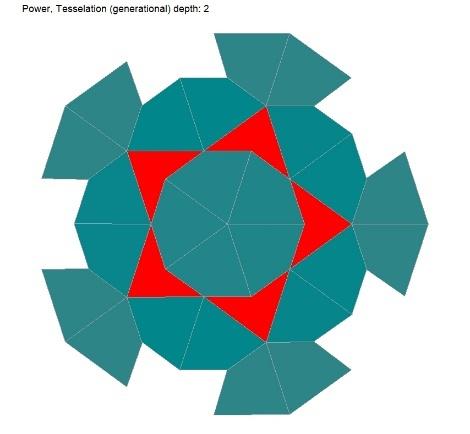

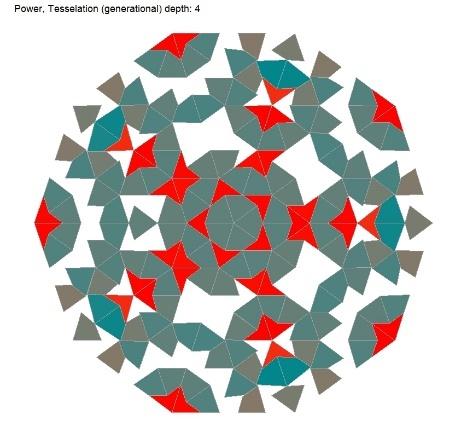

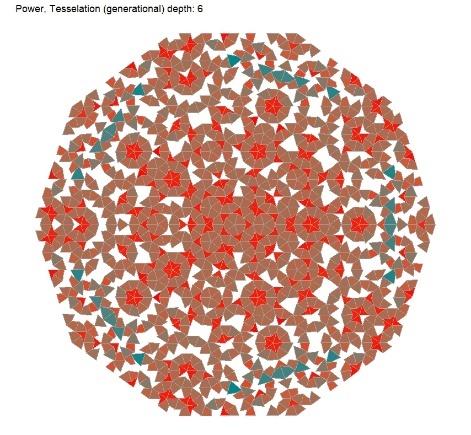


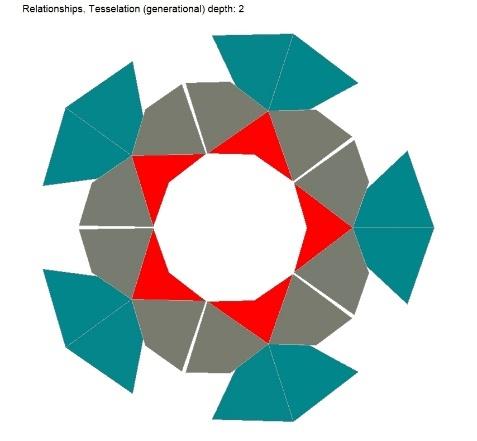

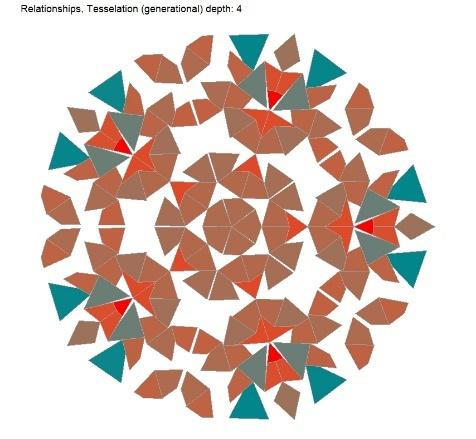

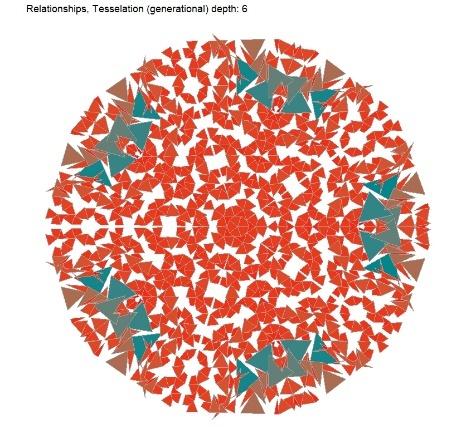


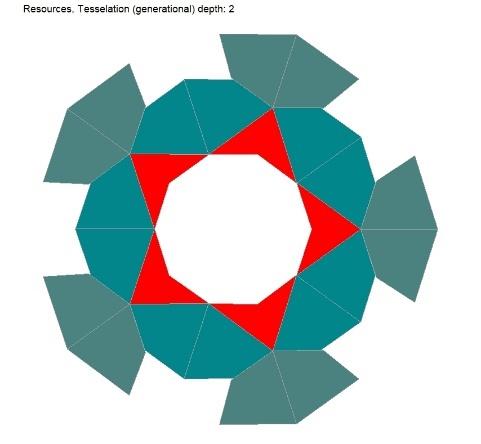

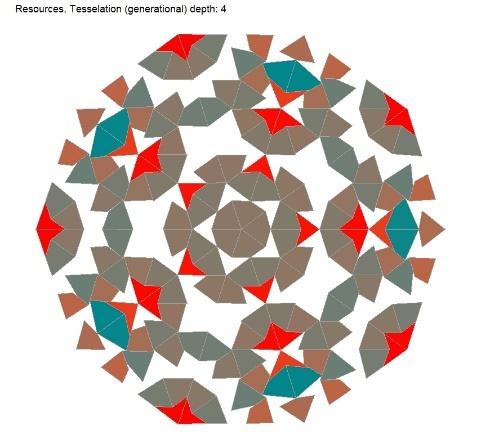

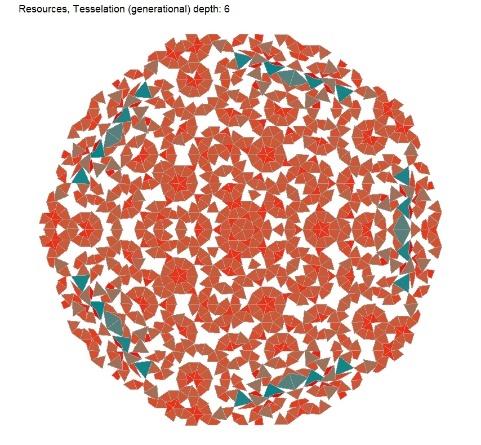


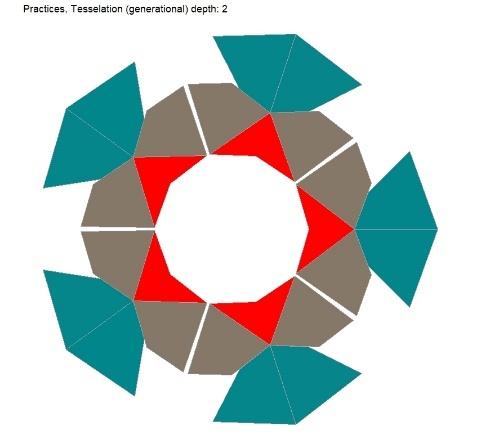

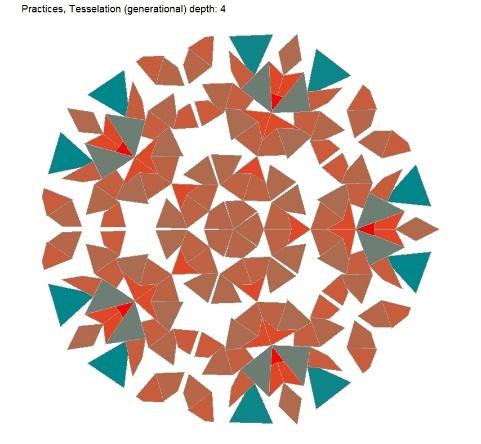

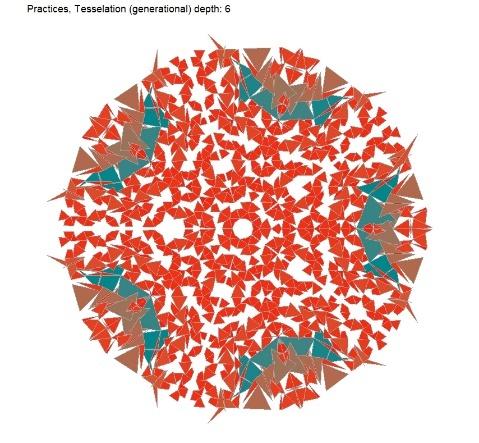


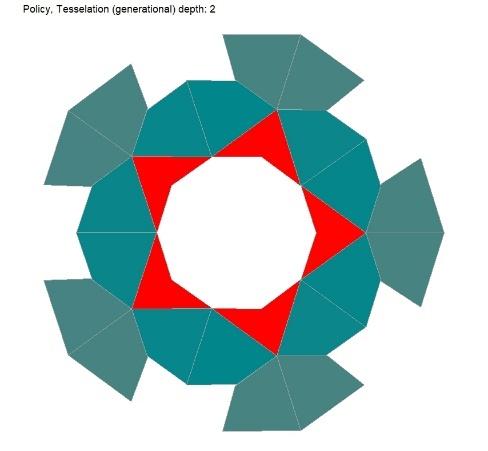

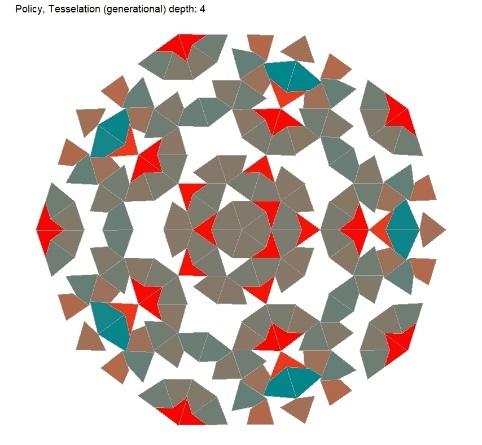

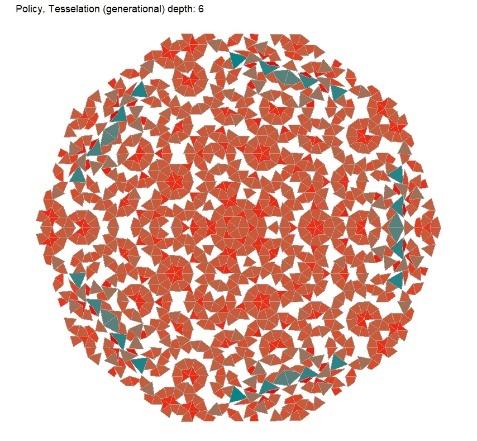


**Appendix B:** Quasi-periodic tessellations generated on each innovation category. We observe how, despite starting off similarly (generation/depth 2 of the tessellation), the patterns diverge eventually (generation/depth 6 of the tessellation), with each progression being self-similar and non-periodic. These final stages help accentuate differences in the original triangles which a static, non-evolving version may not bring out.

***Appendix C: Mathematical technicities of change-point detection.***

With $t_{i}$ representing the time (within the hundred-day period) the i-th individual got relocated, we look at two statistics

$$Z=-2\sum_{i=1}^{n} log\left( \frac{t_{i}}{t_{n}} \right)$$

$$Z_{B}=-2\sum_{i=1}^{n} log\left( 1-\frac{t_{i}}{t_{n}} \right)$$

$Z_{B},$ the backward version, made by switching the flow of time, is the opposite to $Z,$ the forward version. Therefore, a process that deteriorates - that is, one where events (here, relocating people) happen more and more frequently towards the end of the observation period (here 100 days) will inflate the value of ZB and deflate the value of Z. Bhaduri [55] and Ho et al. [54] show how combinations of these two versions

$$R=max\left( Z,Z_{B} \right)$$

$$L=min\left( Z, Z_{B} \right)$$

generate greater power - that is, rightly say more often that a change has occurred when in fact it has, than the individual pieces. A sequence of hypothesis tests controlling for false positive rates [31] called bidirectional tests [43] are conducted through these R and L statistics to pinpoint locations on the time axis, such as the ones shown in red on Figure 5, where drastic shifts from the ongoing rate have likely to have occurred. We point readers interested in further technicalities to [37,43, 45 46,47,48,50,51,52].

1. Interested readers are directed to [53]. [↑](#footnote-ref-1)
