# Supplementary material for: A recipe for systems change: Predictive modeling and street-level bureaucracy among homeless services
Source: PLoS One. 2025 Aug 21;20(8):e0328822. doi: 10.1371/journal.pone.0328822 (PMC12370027; doi:10.1371/journal.pone.0328822)
Supplement: Attachment — Submitted filename: Response to editor and referee comments.docx [file pone.0328822.s003.docx]

Response to editor and referee comments

Dear Authors,

Comment 1: Thank you for submitting your manuscript, "A Recipe for Systems Change: Predictive Modeling and Street-Level Bureaucracy Among Homeless Services." Your efforts to explore the intersection of frontline discretion and predictive modeling in the context of homelessness services is both timely and intellectually ambitious. The paper presents a valuable attempt to combine street-level bureaucracy theory with data-driven analysis and dashboard applications, which is a welcome contribution to both public policy and applied social science.

**Response: We thank the editor for the thoughtful comments and praise. Yes, the above is an apt synopsis of our article. We welcome the feedback, and we feel that the comments provided help improve the manuscript considerably. We thank you and the reviewers for the thoughtful feedback.
We have attached a file with track changes, and we included notes in the document that reference each concern listed here. In doing so, we hope this helps in checking that we have addressed every concern.**

Concern 2: That said, while both reviewers have recommended minor revisions, I believe the manuscript would benefit from more substantial work before it can be considered for publication. In particular, the narrative structure and methodological presentation require refinement to enhance clarity and accessibility for a broader readership.

**Response: Great feedback. As can be seen in the revised copy, particularly in the file with *track-changes*, we have heavily revised the order of the introduction and key sections of the manuscript by paying close attention to narrative flow and clarity. For example, we added section titles, such as “Systems change: A brief synopsis” and “Michael Lipsky’s “street-level bureaucracy” to adequately delineate the topics for easier readability.**

Concern 3: Some sections, especially those covering technical modeling and visual representations, are overly dense or abstract. Readers unfamiliar with the mathematical frameworks may find it difficult to follow, and I encourage you to streamline explanations while maintaining scientific rigor. Consider moving detailed formulaic content or extended metaphors (e.g., shopping carts and recipes) into a supplementary file, and focus in the main text on why the methodology matters in practical terms for systems change and policy implementation.

**Response: Technical discussions have been simplified further by adding explanatory details (shown in the responses below) both in text and in the figure captions. We have separated the mathematical descriptions from the main text and added the following in place:**

*We point readers interested in mathematical technicalities to a crucial appendix (Appendix C) we have designed for this purpose. In summary, we track the times at which homeless people get relocated, construct a number (detailed in appendix C) that serves as the rate of relocations, and used this number to track the earliest instance of a changed rate over the course of the 100 days. Once this earliest instant is found, we begin the algorithm afresh to detect the next possible point(s) of change. Patterns in these detected “change” times hold particular relevance, especially in tracking how time-bound relocation exercises such as the 100-day challenges progress, with and without external (such as governmental) supervision. For instance, with time-bound exercises, a stronger concentration of such change times may be present towards the end of the fixed period which could reflect workers’ upped urgencies. One may compare the efficacy of two time-bound attempts with how similar their change points were, or compare one time-bound study to a general relocation attempt. These “change” times also enable us to measure the impact of a shift. We can contrast the forecasts from the pre-change piece (had there been no change) to the observed relocation numbers from the actual, post-change piece. And possibly ascribe the gap - using field of knowledge - to preventable causes such as absenteeism.* *The strategy described above helps pinpoint where changes occurred, that is, deliver solid definitions of the pre- and post-change pieces, crucial for these gap measurements. We look at two specific examples below to elaborate these matters.*

Concern 4: Tone and language also need attention. Phrases like "throwing money at the problem" and comparisons to "a competent chef" detract from the otherwise academic quality of the manuscript. Please revise for more consistent scholarly language throughout.

**Response: We agree that the language is inflammatory, and the feedback is welcomed. We revised with more academic language for the examples provided here and throughout the paper.**

Concern 5: Additionally, the integration of figures and tables was uneven in the version reviewed. Please ensure all visuals are present, clearly labeled, and thoroughly explained in the text. Some figures, especially those central to the triangle model and dashboard logic, need stronger captions and contextual grounding.

**Response: We apologize for the scant captions on these figures. The captions have now been enlarged to accommodate details as follows:**

Figure 1: Theoretical contributions from Kania et al. (2018).

has been changed to

***Figure 1: Theoretical contributions from Kania et al. (2018). A conceptual triangle is hypothesized which brings out educated guesses on how common certain kinds of changes are likely to occur in relocation exercises. The wider the base, the easier to bring about that change. We notice, therefore, how structural changes are guessed to be easier to bring about than mental changes. We notice, however, two points. First, in this conceptual map, there is no backing from observed data. Second, the cosmetics do not reveal insights: that is, the base or the altitude of the component shapes do not hold special significance. Both these points are taken up in figure 3, a realized version of this conceptual triangle through statistical metrics.***

Previous version of Figure 2: *Frequency of change by theme according to data from RE!NSTITUTE™, based on a theoretical model for “Systems Change” from Kania et al. (2018) using mathematical concepts from Bhaduri (2023).*

has been changed to

***Figure 2: Frequency of change by theme according to data from RE!NSTITUTE™, based on a theoretical model for “Systems Change” from Kania et al. (2018) using mathematical concepts from Bhaduri (2023).*** ***The heights of the towers describe the rate at which changes in the corresponding types were witnessed to occur. For instance, of all the 100-day challenges studied, we found about 55% of times changes in “relationship” were observed, either changes in “relationship” only, or in “relationship” along with those in other types.***

Previous version of Figure 3: *Legitimacy of theoretical model for “Systems Change” from Kania et al. (2018) by theme according to data from RE! RE!NSTITUTE™ and mathematical concepts from Bhaduri (2023).*

has been changed to

***Figure 3: Legitimacy of theoretical model for “Systems Change” from Kania et al. (2018) by theme according to data from RE! RE!NSTITUTE™ and mathematical concepts from Bhaduri (2023).*** ***We interpret this as a realized version on the theoretical triangle shown in figure 1. The shapes and the sizes of the components have been calculated using data from the 100-day challenges. Unlike Figure 1, the bases and the altitudes of the shapes here do convey statistical information. The bases represent how common changes in a certain category are (the wider the base, the more common the change) while the altitudes represent the inverse of the length of the confidence interval for this statistic (the taller the altitude, the more sure we are in the value of the base). This observed empirical triangle (Figure 3), therefore, confirms, in the process, the general shape of the theoretical triangle (Figure 1) in that the bases for “practices”, “policy”, and “resources”, collectively (that is, “structural”) cover the widest ground and the bases subsequently taper down on relational and mental changes.***

Previous version of Figure 4: *A typical map of likely connections that lead to changes in mental structures. The more likely connections are shown in deeper red. We invite readers to explore other possibilities (for instance, those connections that lead to non-mental targets) on our interactive dashboard.*

has been modified to

***Figure 4: A typical map of likely connections that lead to changes in mental structures. The more likely connections are shown in deeper red. We invite readers to explore other possibilities (for instance, those connections that lead to non-mental targets) on our interactive dashboard.*** ***One implements these rules in an input-output sense. If changes in the input details - shown by the blue arrows - are promised, one is given the rate at which changes in the output detail - shown by the red arrow - accompany. The higher this rate, that is, the more (correlationally) automatic the input-output connections are, the darker the shade of the red node representing the rule. For instance, rule 12 says if one designs a relocation study that generates changes in “relationship”, “practice”, “power”, changes in “mental” structures will be promised with a reasonably high chance (50% if one hovers on the dashboard).***

Previous version of Figure 5: *Epochs of stable relocation rates are shown on either side of the red vertical separators for (a) Northern Illinois and (b) Desert City.*

has been changed to

***Figure 5: Epochs of stable relocation rates are shown on either side of the red vertical separators for (a) Northern Illinois and (b) Desert City.*** ***Each vertical separator represents an estimated change-point, that is, a point in time over the course of the 100 days during which the rate of relocating homeless people improved or deteriorated substantially. For instance, had the change-point around day 80 (=400/5) not been there for Northern Illinois, the total number of people relocated could have been 85 instead of 80. Crowding of change points towards the end of the experiment is observed for nearly all regions.***

Concern 6: I also encourage you to strengthen the literature review component. While relevant sources are cited, particularly in relation to Lipsky’s theory, performance measurement tools, and the RE!NSTITUTE framework, these references are scattered throughout the introduction and discussion without a cohesive structure. Consider consolidating and deepening your engagement with prior research in a dedicated section or a more organized narrative format. This will help clarify the theoretical grounding of your work and better situate your contribution within existing scholarly conversations.

**Response: We have revised to lengthen and substantiate the literature review portions of the manuscript, and feel gratitude for this feedback. We feel a sense of gratitude for the opportunity to find more current articles on the topic from the past five to seven years, including one from 2025, which spotlights the relevance of the topic. We feel that this revision helped solidify the topic’s rising importance. You can view these changes at the beginning and end of the section titled Systems change: A brief synopsis and in Michael Lipsky’s “street-level bureaucracy.”
We revised the entire introduction and literature review for clarity and also feel this aided in the revision process of your Concern #2. We feel that the message of the manuscript is much stronger now. Thank you.**

Concern 7: Beyond presentation, I would like to see a fuller discussion of limitations, particularly regarding sample size, generalizability, and potential biases in dashboard design or interpretation. Clarifying the nature of the data (e.g., ethics, consent, and de-identification) would also strengthen transparency.

**Response: We thank the editor for raising this. Limitations have been clarified in the revision in the following way:**

*“While systems change projects can bring about significant benefits, it is crucial to consider the potential for unequal distribution of these benefits. Projects must be designed with equity in mind to ensure that all segments of the population can access and benefit from the changes. This is an interesting avenue to explore as we collect data over future years. Additionally, continuous evaluation and adaptation of these projects are necessary to address emerging challenges and ensure sustained positive outcomes.”*

Regarding **sample size**, we have added:

*“These connections - we stress again - represent correlational links for the moment and need not be taken as causal pathways. In our previous works - Bhaduri (2024), Bhaduri (2025), Camp et al. (2024), Zahirodini and Bhaduri (2025) - we have explored this matter of causality in analogous social settings using similar network structures - Markov random fields - to showcase how gender of respondents and political leanings causally sway their opinion towards current business practices, how students from diverse backgrounds express their sentiments towards the living conditions of migrants at the US-Mexico border, etc. The estimation of these random fields, however, depends on the delicate balance of discerning false positives and false negative edges through regression techniques such as lasso which are data hungry algorithms. In view of the fact that this is the first of a long sequence of ongoing 100-day challenges, data, though of high quality, are relatively scarce, and we have refrained from querying causal effects. In time, as similar high quality data become available, we can deploy the Markov random fields much along the lines of the works cited earlier to explore more interesting causal pathways.”*

Regarding **generalizability,** we have added (in addition to our reply to referee 2's observation on causality - another generalization concern - and the crash example we added now):

*The structural (triangle), correlational (market basket) insights and change point estimates showcased here (the time switched statistic) generalize effortlessly to similar or even more nuanced settings. For example, as of this writing, we are monitoring data coming from the Department of Education and three local school systems in Connecticut to measure the impact of operational tweaks on homelessness programs with special emphasis on the student population. Preliminary results suggest a drop of around 8% in such student population. We are looking at the veteran population in Illinois and New York who may need help in filling out survey forms accurately to stop being moved around in and out of homeless shelters, a separate study in Oregon with sleep trailers, etc. These are instances of shifts in ways of working. These are ongoing studies and are examples of shifts in ways of working (that is, “practice”). We are beginning to notice near simultaneous shifts in relationship among the workers and mental attitudes (but less so in power dynamics and resources) just as the recommendation networks with “Practice LHS” suggests). Generalizability is, thus, evidenced in a potentially nonstationary sense: since the ingredients here are macro properties, so to speak, such as power dynamics or resources which do not fluctuate overnight, recommendation shifts in tendencies are likely to remain valid as time moves. The choice of states, we stress, is not guided necessarily by political or demographic dictates, rather by whichever state authorities partner with the ReInstitute with sound funding and legal agreements. Moving forward, due to the success on display and the quantitative justification backing it, we sense many of the states will be welcoming of these collaborations.*

Regarding **potential biases in dashboard design** (the below) and **interpretations** (the above on causal connections), we have added:

***“Network objects are underexplored in the social sciences. Consequently, the dashboard, which brings out specific kinds of network structures, while extremely insightful, must be interpreted with some caution, in case one is unfamiliar with non-Euclidean mathematics (we direct such readers to Bhaduri (2024), for instance). For instance, contrary to what one typically encounters in a graph, in these structures, there are no horizontal and vertical axes with set roles. As a result, each time one visits the dashboard, there is a slight possibility that this structure may be rotated some different way (although every attempt has been made to prevent this from happening through the Force Atlas algorithm (Setiawan and Bhaduri, 2023)). What matters more, however - the strength of the connections, that is, the rules shown by degrees of red – will stay unchanged. In case a rotated representation results, it is vital, therefore, to acknowledge that the correlation-based recommendations - statements that show changes in which property accompany changes in which others - have not altered. There are other metrics such as the lift ratio through which one may choose to represent the strength of the rules. In the present version of the dashboard, we have refrained from these reports since they will be distracting to the main theme of the current study. With our currently ongoing follow-up studies (under “Future Work”), we are going to supply a variety of such metrics along with suggestions on which might be apt under which circumstance.”***

Concern 8: Finally, please respond in detail to the reviewers’ comments. While they found the work promising, several suggestions, such as clarifying theoretical framing and improving flow—align with the concerns I’ve raised here.

***Response: Yes, we have responded in detail to each concern raised by the reviewers. Please find them below.***

Comment 9: This is a promising paper, and with thoughtful revision, it could make a strong contribution. I invite you to revise the manuscript accordingly and submit a point-by-point response addressing all reviewer and editor feedback.

**Response: We thank the editor again. Especially for the words of encouragement.**

#-----------------Responses to Reviewers’ comments------------------#

Reviewer #1:

Thank you for the invitation to read this interesting and important paper. I find it very well written and coherent. THe theoretical framework and methods are sound. There are some issues that would be possible enhance the author´s argument:

1. some *more theory on systems change than Kania et al should be included*

*2. some more reflections on what is the research gap that the article is addressing should be more explicit and grounded in the state-of-art.*

*3. the discussion lacks a link between results and scientific/theoretical literature.*
**Response**: **We have revised to lengthen and substantiate the literature review portions of the manuscript and feel gratitude for this feedback. We feel this feedback overlaps with the Editor’s Concern 6 (please view our response to Editor’s Concern 6). We also added to the discussion including links between results and scientific/theoretical literature.**

Reviewer #2:

Concern 1: The work is very ambitious to interpret the results derived from the application of both the MBA and network models, as it reflects only associations and not causalities, which reduces the possibility of reliable certainty in decision-making processes.

**Response: We agree with the reviewer’s “associations and not causalities” observation. We humbly point out we noted this towards the end of page 11: “how often mental changes *correlate* with those in relationship..” instead of, say, “mental changes *cause* those in relationship”. Still, as a result of the concern raised, we have chosen to make this point more explicit by adding the following paragraph (right underneath the dashboard link, page 12) which suggests some of our earlier works on Markov random fields that help establish causality, the reason why we chose to refrain from that analysis in the present instance, and how this may be a great future work once we are able to get more data.**

*“**These connections - we stress again - represent correlational links for the moment and need not be taken as causal pathways. In our previous works - Bhaduri (2024), Bhaduri (2025), Camp et al. (2024), Zahirodini and Bhaduri (2025) - we have explored this matter of causality in analogous social settings using similar network structures - Markov random fields - to showcase how gender of respondents and political leanings causally sway their opinion towards current business practices, how students from diverse backgrounds express their sentiments towards the living conditions of migrants at the US-Mexico border, etc. The estimation of these random fields, however, depends on the delicate balance of discerning false positives and false negative edges through regression techniques such as lasso which are data hungry algorithms. In view of the fact that this is the first of a long sequence of ongoing 100-day challenges, data, though of high quality, are relatively scarce, and we have refrained from querying causal effects. In time, as similar high quality data become available, we can deploy the Markov random fields much along the lines of the works cited earlier to explore more interesting causal pathways.”*

**The final part of the comment: “possibility of reliable certainty” is taken care of by the lengths of the confidence intervals, once certainty is interpreted in a correlational sense. The smaller the length, the higher the certainty. This has been stressed in the revision.**

Concern 2: Although it is showed that the geographic issue will be addressed, the text needs to contextualize how projects of this magnitude in such a short period are consistent with the mental changes, practices, and relationships that make a difference, how their construction and continuous strengthening benefits the population, and how the benefits are compared.

**Response: We thank the reviewer for raising this. We have added references in the following paragraphs and reasoning to demonstrate how, even short interventions have the potential to bring about lasting changes, how evaluations are done.**

*“**It may be apt to query whether the mental changes brought about through the 100-day challenge are meant to last. We note here that social change projects, even when implemented over short periods, can substantially influence mental changes, practices, and relationships that benefit the population. These projects often focus on enhancing social security, improving mental health, and fostering social relationships, which are crucial for societal well-being. Social security policies, for instance, have a profound impact on mental health. Changes in social security benefits can influence mental health outcomes such as stress, anxiety, and depression. A systematic review aims to establish this relationship, highlighting the importance of policy in shaping mental health outcomes (Simpson et al., 2020). Short-term interventions, like creative arts therapy programs, have shown significant improvements in children's social behaviors and relationships, indicating that even brief, intensive programs can positively affect mental health and social skills (Buskirk-Cohen, 2015). Organizational change readiness is influenced by social relationships in the workplace. Strong social ties and organizational commitment can facilitate smoother transitions during change, highlighting the importance of fostering supportive environments in social change initiatives (Madsen et al., 2005). Public participation in infrastructure projects can lead to social benefits, although its development is slow. Engaging stakeholders in the planning and execution of projects can enhance social outcomes and address socio-economic disputes (Xie et al., 2014).*

***The construction and continuous strengthening of these projects can lead to sustainable benefits for the population, as they address immediate needs while laying the groundwork for long-term improvements. The benefits of these projects can be compared by examining their impact on mental health, social relationships, and sustainable practices. The benefits of social change projects, such as our 100-day challenge, can be compared by examining their impact on different population groups. For instance, smart mobility policies in the Netherlands showed differential benefits, with car users gaining more than students, raising questions about social equity in policy design (Gironés & Vrščaj, 2018).”***

Comment 3: In the analysis, they address the importance of networks integrated into legal assistance services for homeless people, medical care, and employment services, for example. This could establish other patterns.

**Response: This has been addressed already in Editor’s Concern. Thank you.**

Concern 4: Another issue is replicability and discussing case studies with heterogeneous territorial spaces and social processes from a quantitative perspective, leaving aside the qualitative, which may have very specific implications for the area.

**Response: We acknowledge that applying a standardized quantitative framework to diverse territorial and social contexts can obscure the sociohistorical specificities of each case. As such, we do not claim universal replicability but rather aim to identify patterns that may be indicative rather than definitive. We provide detailed appendices with data sources, coding decisions, and statistical models to support transparency, recognizing that exact replication may be challenged by localized data constraints and social particularities**

Concern 5: MBA algorithms can struggle with large data sets. How did you control this in your model?

**Response: We thank the referee for raising this. In principle, we agree with the comment. In our study, the data size is manageable. A large data set would be a happy problem to have. In anticipation of future waves of data from future runs of the 100-day challenge, we have added the following piece underneath the dashboard link in response to this observation, containing a reference that deals with this precise problem using crash data from large jurisdictions. This should inspire confidence also with the MBA algorithm’s applicability, in general.**

*“Networks - the crucial ingredients in market basket analysis algorithms - are notoriously difficult to estimate and manipulate due to their fundamental non-Euclidean-ness, in case the sample size becomes unmanageably large. While for the current study the sample size is not a concern, we anticipate future waves of 100-day challenge data which could make the algorithm slow or less interpretable. To combat the size problem, we can draw sequential subsamples (while maintaining global network invariants such as the asymmetry of the degree distribution) and create bootstrap intervals to quantify variations in statistics of interest such as the support or the confidence. The approach is becoming standard in the literature. We point interested readers to Pande et al. (2009) where similar algorithms were implemented to fatal crash data in large jurisdictions.”*

Concern 6: The decision on rules for weakness and strength is not explicit in the text.

**Response: This is in line with the Editor’s concern 7. Addressed there.**

Concern 7: With the model used, occurring processes is known, the MBA can act as a “decision aid” by revealing non-obvious patterns, including how these relate to beneficiary behaviors and retention. Or, what are the tools to detect other relationships, such as those related to health? For example, a frontline worker might not realize how frequently substance abuse treatment is related to housing retention unless the data shows it.

**Response: Thank you. The reviewer raises an interesting point. Yes, network-based analysis such as market baskets often bring out non-obvious patterns. Here, in addition to offering quantitative backing to the expected connections, we point out larger, slightly unexpected correlational chains. Some of these are spelled out underneath the dashboard (page 12). Regarding the covariates such as health or substance abuse, we agree those could be potentially vital predictors to guess homelessness (or recurring homelessness). We note the intent behind our analysis was to shed quantitative light on general policy connections instead of deciding whether policy changes would better one specific individual's chances with or without health issues (causal connections here would necessitate the bringing in of A-B tests and uplifts). The point is nonetheless crucial, and taken. We have added the following paragraph. We - the two authors - are gradually becoming more involved in the designing of the questionnaires. We have incorporated some such details (mental or physical state, drug intake, etc.) and we'll be sure to include those results once this initial paper - that lays the foundation - gets published.**

**The chief intent behind the current study was to explore correlational connections between and among working components, and shed quantitative light on the strength of those connections. It was not to decide whether policy changes would better one specific individual's chances with or without health issues, for example (causal connections here would necessitate the bringing in of A-B tests and uplifts). Covariates such as health or substance abuse could be potentially vital predictors to guess homelessness (or recurring homelessness). In future runs of the 100-day challenge, we plan to incorporate these details to offer both population-level and instance-level recommendations.**

Concern 8: Is service fragmentation reduced? Will data prevail over local discretion? Could pattern-based rules lead to rigid or unfair decision-making?

**Response: We are thankful for this feedback and have inserted commentary in the revision accordingly. We’ve labeled in the manuscript where we have done so in the file with track changes, which are at the end of page 21, the bottom of 23 and the top of 24, specifically.**
